# Zanubrutinib, Alone and in Combination With Tislelizumab, for the Treatment of Richter Transformation of Chronic Lymphocytic Leukemia

**DOI:** 10.1097/HS9.0000000000000870

**Published:** 2023-03-24

**Authors:** Constantine Tam, Javier Munoz, Gavin Cull, Stephen Opat, Heather Allewelt, Xiaoping Zhang, Jennifer C. Stern, James Hilger, Kunthel By, Aileen Cohen, Alessandra Tedeschi

**Affiliations:** 1 Alfred Hospital and Monash University, Melbourne, VIC, Australia; 2 Banner MD Anderson Cancer Center, Gilbert, AZ, USA; 3 Sir Charles Gairdner Hospital, Perth, WA, Australia; 4 University of Western Australia, Perth, WA, Australia; 5 Monash Health, Clayton, VIC, Australia; 6 Monash University, Clayton, VIC, Australia; 7 BeiGene, San Mateo, CA, USA; 8 ASST Grande Ospedale Metropolitano Niguarda, Milan, Italy

New therapies for Richter transformation (RT) of chronic lymphocytic leukemia (CLL) are necessary, as current treatments are ineffective for relapsed/refractory disease and prognosis remains poor, particularly for those previously treated for CLL.^1–3^ We report outcomes with the next-generation Bruton tyrosine kinase inhibitor zanubrutinib, alone or in combination with the programmed death-1 inhibitor tislelizumab, from 2 studies.

BGB-3111-AU-003 (NCT02343120) was a 2-part, phase 1/2, open-label study of single-agent zanubrutinib conducted at 24 sites in 6 countries. Part 1 (dose escalation) determined the recommended phase 2 dose (RP2D); part 2 (expansion) evaluated zanubrutinib at the RP2D (160 mg twice daily) in histologic subtypes of B-cell malignancies, including patients with RT (transformation from CLL to diffuse large B-cell lymphoma per World Health Organization classification^4^). All relevant ethics approvals were obtained. The trial was performed in accordance with the Declaration of Helsinki and guidelines for good clinical practice, and all patients provided written informed consent. The methods and results of the part 1 dose escalation have been described previously.^5^ Thirteen patients with locally histologically confirmed RT were enrolled in part 2, with treatment continuing until unacceptable toxicity or disease progression. Median study follow-up was 27.4 months (range, 0.6–33.8 mo) and median duration of zanubrutinib exposure was 18.7 months (range, 0.5–33.8 mo). Patient demographics and baseline disease characteristics are summarized in Table 1.

**Table 1:** Patient Demographics, Baseline Disease Characteristics, and Efficacy Outcomes

	BGB-3111-AU-003 (N = 13)	BGB-3111-A317-001 (N = 7)
Median age, y (range)	69 (54–82)	71 (47–73)
Sex, n (%)		
Male	8 (61.5)	5 (71.4)
Female	5 (38.5)	2 (28.6)
Baseline ECOG performance status, n (%)		
0	5 (38.5)	3 (42.9)
1	6 (46.2)	2 (28.6)
2	2 (15.4)	2 (28.6)
Bulky disease (LDi ≥10 cm), n (%)	0	2 (28.6)
Bone marrow involvement, n (%)	8 (61.5)	4 (57.1)
Extranodal disease, n (%)^*a*^	10 (76.9)	4 (57.1)
Treatment-naive, n (%)^*b*^	1 (7.7)	0
Refractory disease, n (%)^*c*^	8 (61.5)	7 (100)
Baseline cytopenia, n (%)^*d*^	7 (53.8)	0
Number of therapies before histologic confirmation of RT, median (range)	1 (0–5)	Not available
Therapies received before histologic confirmation of RT, n (%)		
Anti-CD20-based regimen	8 (61.5)	Not available
CHOP or R-CHOP	3 (23.1)	
Bendamustine/rituximab	2 (15.4)	
Ibrutinib	1 (7.7)	
Number of prior therapies following histologic confirmation of RT, median (range)	1 (0–3)	3 (1–5)
Therapies received following histologic confirmation of RT, n (%)		
Anti-CD20-based regimen	7 (53.8)	7 (100)
CHOP or R-CHOP	8 (61.5)	6 (85.7)
Bendamustine/rituximab	1 (7.7)	0
Ibrutinib	2 (15.4)	0
Prior radiation therapy, n (%)	3 (23.1)	1 (14.3)
Prior stem cell transplant, n (%)	1 (7.7)^*e*^	0
Median study follow-up time, mo (range)	27.4 (0.6–33.8)	15.4 (3.5–51.5)
Overall response, n (%) [95% CI]	8 (61.5) [31.6-86.1]	3 (42.9) [9.9-81.6]
Complete response, n (%) [95% CI]	2 (15.4) [1.9-45.4]	1 (14.3) [0.3-57.8]
Median duration of response, mo (range)	25.4 (0+ to 29.7+)	17.2 (2.9–48.4)
Median progression-free survival, mo (range)	17.3 (0.6–32.2+)	2.9 (2.4–51.4+)
Median overall survival, mo (range)	29.3 (0.6–33.8+)	15.4 (3.5–51.5+)

“+” indicates censored observation.

^*a*^Extranodal disease is defined as patients with extranodal baseline target or nontarget lesions, or bone marrow involvement by biopsy, per investigator assessment.

^*b*^Disease status at study entry (treatment-naive, relapsed, refractory) refers to RT and not the CLL from which it transformed.

^*c*^Refractory disease is defined as best overall response of stable disease or progressive disease from last prior anticancer treatment regimen.

^*d*^Cytopenia is defined as baseline neutrophils ≤1.5 × 10^9^/L, baseline platelets ≤100 × 10^9^/L, or baseline hemoglobin ≤110 g/L.

^*e*^This patient underwent stem cell transplantation 43 mo before enrollment in this trial.

CI = confidence interval; CHOP = cyclophosphamide, hydroxydaunorubicin hydrochloride, vincristine, and prednisone; CLL = chronic lymphocytic leukemia; ECOG = Eastern Cooperative Oncology Group; LDi = longest diameter of lymph nodes; R-CHOP = rituximab, cyclophosphamide, hydroxydaunorubicin hydrochloride, vincristine, and prednisone; RT = Richter transformation.

All 13 patients experienced at least 1 treatment-emergent adverse event (AE); 9 (69.2%) experienced at least 1 grade 3 AE. Nine (69.2%) patients had infection AEs; none were opportunistic infections. Cytopenia AEs included thrombocytopenia (4 [30.8%] patients), anemia (3 [23.1%] patients), and neutropenia (2 [15.4%] patients). Six (46.2%) patients developed serious AEs, with retroperitoneal mass, acute myocardial infarction, and bacteremia leading to treatment discontinuation in 3 (23.1%) of them. Atrial fibrillation/flutter and tumor lysis syndrome (TLS) AEs were not observed. Two patients died from AEs (myocardial infarction and bacteremia). Eight (61.5%) patients achieved response by computed tomography (CT) or positron emission tomography (PET)/CT per Lugano classification^6^ (complete response [CR], 2 [15.4%] patients; partial response [PR], 6 [46.2%] patients; Table 1). One of the 3 patients who received prior ibrutinib for CLL did not respond to zanubrutinib treatment. Two patients received prior ibrutinib for RT: 1 achieved a PR lasting 76 weeks; 1 died of myocardial infarction before the first response assessment. Estimated median duration of response (DOR) was 25.4 months (95% confidence interval [CI], 5.9 to not estimable). Two patients were not evaluable for efficacy: 1 patient died before the first tumor assessment; the other had bone marrow involvement and was not evaluable due to absence of a target lesion at study entry, but the patient remained well without disease progression at the end of study and enrolled in the long-term extension (LTE) study (BGB-3111-LTE1 [NCT04170283]) to continue treatment with zanubrutinib. Of the 8 patients with partial or better responses, 6 responded for ≥12 months, 3 for ≥24 months, and 1 had an ongoing response at 29 months; none reported initiation of subsequent therapy before cessation of response. Four patients with continued response remained on zanubrutinib treatment at study end and were enrolled in the LTE study. Response was maintained at their last LTE visit, with DOR of ≥28, ≥36, ≥38, and ≥42 months. Figure 1 shows progression-free survival (PFS). Estimated median PFS was 17.3 months (95% CI, 2.8 to not estimable). One patient was event-free for ≥32.2 months at final disease assessment before study discontinuation. No patients underwent subsequent allogeneic transplant during the study follow-up period.

**Figure 1.**
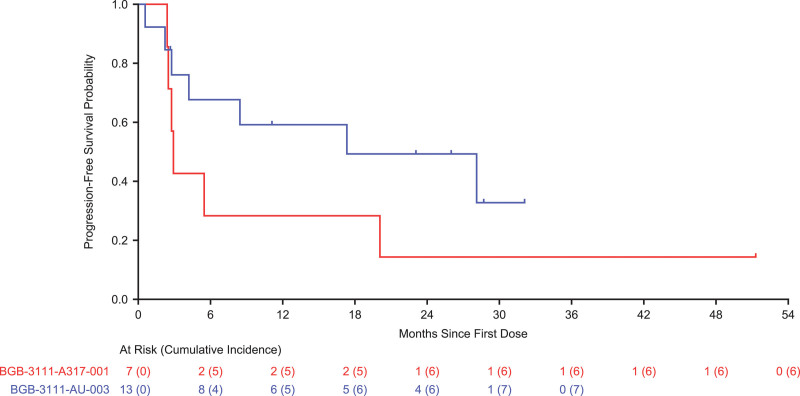
**Kaplan-Meier plot of investigator-assessed progression-free survival in RT patients in each study.** Patients in Study BGB-3111-AU-003 received zanubrutinib monotherapy. Patients in Study BGB-3111-A317-001 received zanubrutinib in combination with tislelizumab. Vertical lines indicate censored observation. RT = Richter transformation.

BGB-3111-A317-001 (NCT02795182) was a 2-part, phase 1/2, open-label study (13 sites in Australia and China) evaluating the safety and preliminary efficacy of zanubrutinib in combination with tislelizumab in patients with B-cell lymphoid malignancies, including RT. Part 1 (dose escalation) determined the RP2D; part 2 (expansion) evaluated the 2 drugs at the RP2D (tislelizumab 200 mg every 3 weeks; zanubrutinib 160 mg twice daily). Treatment continued in 21-day cycles until unacceptable toxicity or disease progression. All relevant ethics approvals were obtained. The trial was performed in accordance with the Declaration of Helsinki and guidelines for good clinical practice, and all patients provided written informed consent. Seven patients with relapsed RT were enrolled in the study. Median follow-up was 15.4 months (range, 3.5–51.5 mo). Patient demographics and baseline disease characteristics are summarized in Table 1.

All 7 patients experienced at least 1 AE; 5 experienced grade ≥3 AEs, most commonly neutropenia (4 patients), platelet count decrease (2 patients), and TLS (3 patients). All 3 cases of TLS were grade 3, nonserious, and resolved within 5 days. One patient developed TLS on day 2 of treatment with zanubrutinib (tislelizumab not begun) and received allopurinol. Two patients developed TLS later, on days 131 and 188 of combination therapy, which were deemed unrelated to study drugs by the investigators; 1 received rasburicase. Five patients developed serious AEs, of which 4 (drug hypersensitivity reaction, shortness of breath, pneumonitis, and fractured femur) led to study discontinuation. No serious AEs led to death.

Three (42.9%) patients achieved response by CT or PET/CT per Lugano classification^5^ (CR, 1 [14.3%] patient; PR, 2 [28.6%] patients; Table 1) and responded for 17.5 months (CR), 2.9 months (PR), and ≥48.4 months (PR), respectively. Figure 1 summarizes PFS. Five patients progressed before 6 months, 1 progressed at 20 months, and 1 was event-free for ≥51 months. The latter prolonged response was particularly remarkable as study treatment had been discontinued at 24 months due to grade 3 immune-related pneumonitis, yet having received no further antineoplastic therapy, the patient remains progression-free as of last follow-up in the LTE study. No patients underwent subsequent allogeneic transplant during the study follow-up period.

Emerging therapies have been evaluated in similarly small patient populations with RT (4–23 patients); they include ibrutinib, alone or in combination with methylprednisolone^7^ or nivolumab,^8,9^ as well as single-agent therapy with venetoclax,^10^ selinexor,^11^ and pembrolizumab.^2,12^ Overall response rates (ORR) of 13%–75% have been recorded with these therapies. In a study of acalabrutinib monotherapy in 25 patients with RT,^13^ ORR was 40% and median PFS was 3.2 months,^13^ which was substantially lower than with the zanubrutinib monotherapy reported here. Recently presented data from a study of pirtobrutinib monotherapy, which included 57 patients with RT (50 response evaluable), demonstrated promising efficacy, with an ORR of 54% and median DOR of 8.6 months.^14^ Due to the small sample sizes in the current analysis, it cannot be concluded that zanubrutinib is more effective for RT as monotherapy than in combination with tislelizumab. One possible reason for the difference in outcomes between the 2 studies is that 100% of patients in the BGB-3111-A317-001 combination study had refractory disease at study entry, compared with 61.5% in BGB-3111-AU-003. Other limitations of both studies, which should be highlighted, are that RT diagnosis was made by local pathology evaluation and molecular analyses (of either RT or preceding CLL) were not performed centrally nor were data from local molecular analyses required. As RT from CLL is often a complex diagnosis, and as outcomes may be impacted by these factors, it will be important to include these evaluations in future trials.

Data from BGB-3111-AU-003 and BGB-3111-A317-001 demonstrate that zanubrutinib has antineoplastic activity in patients with RT from CLL. Advantages of combination with tislelizumab, as with other PD-1 inhibitors, require confirmation from additional studies. While cross-trial comparisons cannot be made, the CR rate of ~15% in patients with RT from each study and sustained responses in some patients are encouraging and warrant further investigation.

## ACKNOWLEDGMENTS

The authors thank the patients who participated in the study, their supporters, and the investigators and clinical research staff from the study centers. Editorial assistance was funded by BeiGene and provided by Ify Sargeant, DPhil, and Holly Strausbaugh, PhD, of Twist Medical.

## AUTHOR CONTRIBUTIONS

HA developed the first draft of the letter. All authors made substantial contributions to the execution of the study, were involved in the analysis and interpretation of the data, reviewed and substantively revised the letter, and approved the letter for submission.

## DATA AVAILABILITY

On request, and subject to certain criteria, conditions, and exceptions, BeiGene will provide access to individual de-identified participant data from BeiGene-sponsored global interventional clinical studies conducted for medicines (1) for indications that have been approved or (2) in programs that have been terminated. Data requests may be submitted to: DataDisclosure@beigene.com.

## DISCLOSURES

CT reports honoraria and institutional research funding from BeiGene, Janssen, and AbbVie. JM reports consulting for Pharmacyclics/AbbVie, Bayer, Gilead/Kite Pharma, Pfizer, Janssen, Juno/Celgene, BMS, Kyowa, Alexion, Fosunkite, Innovent, Seattle Genetics, Debiopharm, Karyopharm, Genmab, ADC Therapeutics, Epizyme, BeiGene, Servier, Novartis, and Morphosys/Incyte; research funding from Bayer, Gilead/Kite Pharma, Celgene, Merck, Portola, Incyte, Genentech, Pharmacyclics, Seattle Genetics, Janssen, and Millennium; honoraria from Targeted Oncology, OncView, Curio, Kyowa, Physicians’ Education Resource, and Seattle Genetics; and serving on speakers’ bureaus for Gilead/Kite Pharma, Kyowa, Bayer, Pharmacyclics/Janssen, Seattle Genetics, Acrotech/Aurobindo, BeiGene, Verastem, AstraZeneca, Celgene/BMS, and Genentech/Roche. GC reports research funding from BeiGene, AstraZeneca, and LOXO Oncology; and honoraria/travel support from Roche and Glycomimetics. SO has acted as a consultant/advisor for AbbVie, BeiGene, Janssen, Gilead, Roche, Mundipharma, Merck, and Bristol Myers Squibb; has received research funding from AbbVie, BeiGene, Janssen, Gilead, Roche, and Epizyme; and has received honoraria from AbbVie, BeiGene, Janssen, Gilead, Roche, Merck, and Bristol Myers Squibb. HA, XZ, JS, and JH, are employees of, and own stock in, BeiGene. KB is an employee of BeiGene. AT reports serving on advisory boards and speakers’ bureaus for AbbVie, Janssen Spa, AstraZeneca, and BeiGene.

## SOURCES OF FUNDING

This study was supported by research funding from BeiGene (Beijing) Co., Ltd., Beijing, China. This work was supported by BeiGene USA, Inc.
